# The Relevance of Arterial Blood Pressure in the Management of Glaucoma Progression: A Systematic Review

**DOI:** 10.1093/ajh/hpad111

**Published:** 2023-11-23

**Authors:** Jan Van Eijgen, Jesus D Melgarejo, Jana Van Laeken, Claire Van der Pluijm, Hanne Matheussen, Micheline Verhaegen, Karel Van Keer, Gladys E Maestre, Lama A Al-Aswad, Thomas Vanassche, Zhen-Yu Zhang, Ingeborg Stalmans

**Affiliations:** Department of Ophthalmology, University Hospitals UZ Leuven, Leuven, Belgium; Research Group Ophthalmology, Department of Neurosciences, KU Leuven, Leuven, Belgium; Institute of Neurosciences, School of Medicine, University of Texas Rio Grande Valley, Harlingen, Texas, USA; Rio Grande Valley Alzheimer’s Disease Resource Center for Minority Aging Research (RGV AD-RCMAR), University of Texas Rio Grande Valley, Brownsville, Texas, USA; Department of Ophthalmology, University Hospitals UZ Leuven, Leuven, Belgium; Research Group Ophthalmology, Department of Neurosciences, KU Leuven, Leuven, Belgium; Research Group Ophthalmology, Department of Neurosciences, KU Leuven, Leuven, Belgium; Department of Ophthalmology, University Hospitals UZ Leuven, Leuven, Belgium; Research Group Ophthalmology, Department of Neurosciences, KU Leuven, Leuven, Belgium; Research Group Ophthalmology, Department of Neurosciences, KU Leuven, Leuven, Belgium; Institute of Neurosciences, School of Medicine, University of Texas Rio Grande Valley, Harlingen, Texas, USA; Rio Grande Valley Alzheimer’s Disease Resource Center for Minority Aging Research (RGV AD-RCMAR), University of Texas Rio Grande Valley, Brownsville, Texas, USA; Department of Human Genetics, School of Medicine, University of Texas Rio Grande Valley, Brownsville, Texas, USA; Department of Ophthalmology, New York University (NYU) School of Medicine, NYU Langone Health, New York, USA; Centre for Molecular and Vascular Biology, Department of Cardiovascular Sciences, KU Leuven, Leuven, Belgium; Research Unit Hypertension and Cardiovascular Epidemiology, Department of Cardiovascular Sciences, KU Leuven , Leuven, Belgium; Department of Ophthalmology, University Hospitals UZ Leuven, Leuven, Belgium; Research Group Ophthalmology, Department of Neurosciences, KU Leuven, Leuven, Belgium

**Keywords:** 24-h ABPM, blood pressure, blood pressure variability, glaucoma, hypertension, nocturnal dipping

## Abstract

**BACKGROUND:**

Glaucoma is one of the leading causes of global blindness and is expected to co-occur more frequently with vascular morbidities in the upcoming years, as both are aging-related diseases. Yet, the pathogenesis of glaucoma is not entirely elucidated and the interplay between intraocular pressure, arterial blood pressure (BP) and ocular perfusion pressure is poorly understood.

**OBJECTIVES:**

This systematic review aims to provide clinicians with the latest literature regarding the management of arterial BP in glaucoma patients.

**METHODS:**

A systematic search was performed in Medline, Embase, Web of Science and Cochrane Library. Articles written in English assessing the influence of arterial BP and systemic antihypertensive treatment of glaucoma and its management were eligible for inclusion. Additional studies were identified by revising references included in selected articles.

**RESULTS:**

80 Articles were included in this systemic review. A bimodal relation between BP and glaucoma progression was found. Both high and low BP increase the risk of glaucoma. Glaucoma progression was, possibly via ocular perfusion pressure variation, strongly associated with nocturnal dipping and high variability in the BP over 24 h.

**CONCLUSIONS:**

We concluded that systemic BP level associates with glaucomatous damage and provided recommendations for the management and study of arterial BP in glaucoma. Prospective clinical trials are needed to further support these recommendations.

Glaucoma is one of the leading causes of visual impairment and blindness worldwide.^1–3^ The disease is characterized by structural and functional damage of the optic nerve head due to progressive loss of retinal ganglion cells and their axons.^4,5^ High intraocular pressure (IOP) is a major risk factor for disease development and progression. Other risk factors include age, family history, ethnicity, diabetes, and myopia.^1,6–10^ The rates of both glaucoma and high IOP are expected to co-occur more frequently as their rates keep rising parallel to the increasing life expectancy.^1,11^ To date, IOP is the only modifiable risk factor and therapeutic option in glaucoma. Nevertheless, some patients with normal or well-controlled IOP are still at risk for glaucomatous damage. The vascular paradigm suggests impaired systemic vascular function, thus compromising blood supply to the optic nerve head, as a risk factor for disease progression.

The interplay between IOP, blood pressure (BP), and ocular perfusion pressure (OPP) is poorly understood which limits the development of a universal consensus around these parameters in the management of glaucoma.^1,9^ The exact interaction between the course of glaucoma and arterial BP is complex with studies supporting both arterial hypotension and hypertension as protective and risk factors.^1,4–6,8,11–14^ The role of BP in relation to glaucoma risk has been clarified by the use of 24-h ambulatory BP monitoring. Nocturnal hypotension is considered a potential systemic vascular risk factor for glaucoma. Moreover, abnormal circadian rhythms—such as an increased nighttime BP, an absence of nocturnal BP dipping, or an excessive nocturnal BP dip—have been associated with target-organ damage and increased cardiovascular risk.^1,2,13,15^ However, even with the cumulative evidence on dysregulations in BP, evidence-based clinical decision-making remains challenging due to conflicting evidence and the poor understanding of the complex interplay between glaucoma and BP. To address these challenges, we performed a systematic review of studies evaluating the role of BP and antihypertensive medication in glaucoma.

## METHODS

The Preferred Reporting Items for Systematic Reviews and Meta-Analyses (PRISMA) statement was used as a guidance for this review.^16^

### Eligibility criteria

Articles that assessed the association between arterial BP and glaucoma were included. Other inclusion criteria were: (i) articles evaluating arterial BP in glaucoma patients, (ii) articles assessing the impact of BP on IOP and parameters of progression such as retinal nerve fiber layer changes, ganglion cell layer changes, visual field defects, and optic disc hemorrhages, and (iii) articles evaluating the impact of antihypertensive medication in glaucoma patients/on glaucoma progression.

We excluded articles that (i) already feature in meta-analyses in this review, (ii) articles that only reported on glaucoma prevalence in hypertensive cohorts. Other exclusion criteria were: (iii) a non-glaucoma population or animal studies, (iv) meeting abstracts and conference proceeding, and (v) articles written in other languages than English.

### Search strategy

Articles were identified by searching Medline (via PubMed), Embase, Web of Science and Cochrane Library. The three concepts “glaucoma,” “arterial blood pressure,” and “management” and their synonyms were combined to search several electronic databases. The “carrot²” search results clustering engine’ was used to broaden the concept “management.” After reference import and deduplication selection based on title, abstract, and full text was executed respectively. The search was last performed on 28-07-2022 by researchers JVL and JVE and inconsistencies were solved by consensus. Replies on included articles were included to allow critical appreciation by the scientific community but are listed separately. Relevant articles found by scanning reference lists of included articles but published before 2015 were included only when they were cited in multiple included articles.

The following search terms were used in Medline (Pubmed): glaucoma*, arterial pressure*, arterial tension, artery pressure, intraarterial pressure, blood pressure*, hypertensi*, hypotensi*, Disease Management, Management*, Monitoring, therap*, treatment, adapt*, change*, approach*, disease control*, risk*, progression*, visual field*. Filters “English,” “2015–2022,” and “full article” were applied. Animal studies were exclude using search blocks. The full search strategy, as well those for the other databases, can be found under Supplementary Data.

## RESULTS

### Study selection

The search of five electronic databases provided a total of 8,681 citations. After deduplication, 6,581 references remained. Of these, 6,276 were excluded based on title and 181 studies were excluded based on abstract, since these papers did not meet the inclusion criteria. Full texts of the remaining 124 papers were thoroughly examined. It appeared that 28 studies did not meet the previously described inclusion criteria. Seven additional citations were identified by searching reference lists of relevant papers.

### Synthesis of results

#### Arterial hypertension

First, the relationship between hypertension and IOP is straightforward. It is hypothesized that high BP increases IOP by a dual mechanism. First high BP increases the blood flow and capillary perfusion pressure in the ciliary body leading to an increased production of aqueous humor. Second, high BP, decreases the aqueous outflow through an elevated episcleral venous pressure.^1,17^ Generally, for every 10 mm Hg increase in BP there is a ca. 0.28 (0.08–0.48) mm Hg increase in IOP.^1,9,17–19^

Large-scale epidemiologic studies in the past aimed to explain the relationship between hypertension and glaucoma, with limited clinical implications. On the one hand, hypertension and increased blood flow leading to an increased OPP could compensate elevated IOP. On the other hand, among chronic hypertensive patients, progressive endothelial dysfunction through hypertensive microvascular damage compromises this positive effect on OPP resulting in suppressed endothelial vasoreactivity, hypoperfusion of the optic nerve head and progressive glaucomatous neurodegeneration. Of note, measurement of the absolute OPP does not exist, therefore arterial BP is used as a proxy measure.^4,6,10,20–26^ Several studies evaluating multiple systemic risk factors including hypertension, as well as a genome-wide association mendelian meta-analysis, could not find a statistically significant effect of hypertension on glaucoma progression.^27–30^ However, most studies, including a recent meta-analyses of 16 studies, showed that hypertension is a risk factor for the development and progression of glaucoma (cfr. Table 1).^31–42^ A 2020 meta-analysis concluded that (mostly office) hypertension, next to non-physiological BP dipping, was the most significant risk factor for primary open-angle glaucoma (POAG) among the evaluated systemic vascular factors.^7^

**Table 1:** Arterial hypertension

		Type of study	Study population	Patients	Eyes	Men/women	Country/ Ethnicity	Age	Hypertension	Antihypertensives	Conclusion	Significance level
Dielemans et al.^33^	1995	Single-center prospective cohort study	POAG and NTG	4,187	8,374	1,662/2,525	The Netherlands	55–95 years	n.s.	1,747	POAG was significantly associated with SBP and arterial hypertension, NTG was not	*P* < 0.05
Mitchell et al.^39^	2004	Population-based cohort study	Residents in an area West of Sydney	3,654	7,308	n.s.	Australia	49–97 years	1,669	Included	Systemic hypertensive subjects, especially those with poorly controlled hypertension, had a higher risk of glaucoma, independent of other risk factors	*P* < 0.05
Gangwani et al^44^.	2015	Prospective population-based study	Patients treated with systemic antihypertensives	110	n.s.	64/46	China	65.1 ± 9.5 years	All patients	All patients	NTG was the most prevalent glaucoma subtype. Higher SBP, DBP and MAP were associated with thinner RNFL thickness. MAP was positively correlated with IOP	*P* < 0.05
Actis et al.^31^	2016	Retrospective, observational study	POAG	190	377	76/114	Caucasian	61.49 ± 9.58 years	n.s.	n.s.	Among other things systemic hypertension was statistically significant associated with worsening of the MD variable (*P* < 0.0001)	n.s.
Feraru et al.^28^	2016	Retrospective study	POAG	69	69	16/53	Romania	Mean 62.3 years	39	n.s.	Arterial hypertension was not significantly associated with glaucoma progression. Progression rate was only correlated with the initial and final MD level	*P* < 0.05
Rim et al.^41^	2017	Retrospective propensity-score-matched cohort study	Systemic hypertensive patients on anti-hypertensive medication and normotensive controls	200,124(100,062 + 100,062)	n.s.	129,577/ 70,547	Korean	>40 years	100,062	100,062	Systemic hypertension was associated with an 1.16-fold increased risk for POAG development. Hypertensive patients <65 years were more susceptible to POAG (HR = 1.17)	*P* < 0.05
Kosior-Jarecka et al.^45^	2017	Retrosprective study	NTG	215	280	64/151	Caucasian	70.5 ± 10 years	104	n.s.	Systemic hypertension was 2 times more frequently observed in NTG patients with arcuate scotoma (*P* < 0.001)	*P* < 0.05
Chan et al.^27^	2017	Retrospective case-control study	Rapidly and non-rapidly progressing glaucoma	534 (48 + 486)	540 (54 + 486)	227/313	Australia	Rapid progressors: 83 ± 9.83 years Non-rapid progressors: 79 ± 10.63 years	339	n.s.	Systemic hypertension was not a statistically significant risk factor for rapid progression (*P* = 0.22)	*P* < 0.05
Khatri et al.^43^	2018	Hospital-based, cross-sectional descriptive study	POAG	221	442	107/114	Nepal	54.4 ± 15.9 years	81	All patients with hypertension	Patients with arterial hypertension, diabetes mellitus or both have significantly more severe POAG (based on anatomical and functional loss) and could represent “high-risk patients” with POAG (OR 2,75, *P* = 0.001)	*P* < 0.05
Cantor et al.^20^	2018	Cross-sectional study	POAG and controls (non-glaucoma) with treated systemic hypertension	1,272 (196 + 1,076)	n.s.	443/829	Colombia	≥50 years	All patients	All patients	High values of diastolic BP (>90 mm Hg) and low values of OPP (<40 mm Hg) and DPP (≤50 mm Hg) were associated with a ± two times higher risk of confirmed POAG. These relationships were not modified by the type of AHT	*P* < 0.05
Krishnan et al.^56^	2018	Descriptive study	NTG	41	81	15/26	India	51.75 ± 10.91 years	13	n.s.	Arterial hypertension is an important risk factor for NTG. A DPP <50 mm Hg was statistically significant inverse correlated with the VFD	*P* < 0.05
Hussain et al.^8^	2019	Hospital-based cohort study	POAG cases and suspects	100	n.s.	n.s.	Pakistan	≥50 years	All patients	All patients were treated with AHT for at least 1 year before the inclusion	DBP >90 mm Hg was associated with increased IOP and a 2.2 times higher risk to have confirmed POAG (*P* = 0.08). The type of AHT did not modify this relationship	*P* < 0.05
Gore et al.^55^	2019	Hospital-based, case control cross-sectional observation study	OAG and controls	150 (75 + 75)	150	84/66	India	30–80 years	n.s.	Excluded	Conventional defined systemic hypertension and OAG were not associated. DPP <55 mm Hg and OPP <50 mm Hg are significantly associated with a 5–6 times (respectively) increased risk for POAG	*P* < 0.001
Kuang et al.^36^	2020	Case-control study	POAG and controls	562,300 (112,929 + 449,840)	n.s.	296,145/ 266,155	Han-Chinese	Average 59 years	296,975	296,975	POAG was, among other things, significantly associated with prior systemic hypertension (*P* < 0.001)	*P* ≤ 0.05
Park et al.^25^	2020	Cross-sectional retrospective study	POAG and controls	103 and 58	179 and 92	41.8% and 40.2%	South Corea	58.5 and 52.6	31.8% and 18.6%	n.s.	In POAG participants with disc hemorrhage, high BP was associated with reduction in macular vessel density	*P* = 0.003
Dascalu et al.^32^	2020	Prospective cohort study	OAG	102		139	Romania	51	35	n.s.	Glaucoma progression was associated with systemic vascular risk factors including diastolic low BP, ischemic cardiac disease, peripheral vasospasm, and hypertension	
Marshall et al.^38^	2021	Prospective, longitudinal study	Early manifest POAG	1,222	2,444	601/621	Australia	63.9 ± 11.1 years	467	Included	Systemic hypertension and AHT were significantly associated with both structural (*P* = 0.006 and *P* = 0.010, respectively) and functional (*P* = 0.013 and *P* = 0.010) progression	*P* < 0.05
Ch’ng et al.^21^	2021	Prospective cohort study	POAG, NTG, and PCAG	164	164	43/17, 30/22, and 19/33		63.0 ± 9.4, 59.6 ± 10.3, and 62.3 ± 8.5	44, 38, and 29	Included	Moderate to severe glaucomatous optic damage was associated with lower systolic and diastolic BP	*P* < 0.05
Gillespie et al.^35^	2021	Clinical trail registry	POAG	1,118	1,118	330/269 and 240/279		57.9 ± 0.9 and 65.3 ± 9.3	37	486	Office hypertension significantly associates with slope changes in visual field defects, with estimates ranging from −0.33 dB/year to −0.18 dB/year	*P* < 0.05
Funk et al.^34^	2022	Retrospective case-control study	NTG and healthy controls	277	277	159/118 in both	United States (white, asian, black, hispanic, native american)	69.5 ± 10.5 and 68.9 ± 10.0	181 and 148	315 and 266	Patients with NTG had significantly higher rates of systemic hypertension (OR, 1.64; *P* = 0.004)	*P* < 0.05
Changet et al. ^29^	2022	Prospective, longitudinal study	POAG	119	191	57/62	USA (European or African ancestry)	66.3 ± 10.3 years	44	n.s.	No significant OCT or VF progression rate difference between hypertensive and non-hypertensive patients	n.s.
Plotnikov et al.^30^	2022	Genome-wide association study meta-analysis	POAG and controls	70,832	n.s.	n.s.	European ancestry	n.s.	n.s.	n.s.	Mendelian randomization analysis did not support a causal relationship of BP on IOP change or POAG prevalence	*P* < 0.05

**Abbreviations**: OAG: open-angle glaucoma; POAG: primary open-angle glaucoma; NTG: normal-tension glaucoma; SBP: systolic blood pressure; DBP: diastolic blood pressure; MAP: mean arterial pressure; RNFL: retinal nerve fiber layer; IOP: intraocular pressure; MD: mean deviation; HR: hazard ratio; OPP: ocular perfusion pressure; DPP: diastolic perfusion pressure; AHT: antihypertensive treatment; VFD: visual field defect; n.s.: not specified

In accordance, a Nepalese study with 221 hypertensive POAG patients confirmed this finding. Patients with office hypertension, diabetes mellitus or the combination of both had a higher severity of POAG, with an odds ratio for severe visual field defects of 2.75, 4.72, and 19.9 (*P* = 0.001, *P* = 0.0031, and *P* = 0.0046); respectively. In this study, IOP did not differ between those with or without arterial hypertension.^43^ In 2015 a cross-sectional study found that higher systolic BP, diastolic BP and mean arterial pressure (MAP) were associated with thinner retinal nerve fiber layer thickness. They also observed a positive correlation between MAP and IOP.^44^

A study regarding normal-tension glaucoma (NTG) patients found that systemic hypertension was two times more frequently observed in NTG patients with arcuate scotomas. They hypothesized that the systemic vascular profile of the patient could predict the morphology of early scotoma in NTG.^45^

#### Circadian BP dysregulations

##### Nocturnal hypotension

Systemic BP is a dynamic parameter that follows a normal circadian rhythm. During the night, BP physiologically decreases between 10% and 20% compared to the daytime BP level, this is categorized as normal dipping.^46^ However, in certain conditions, the nighttime BP either decreases too much (extreme dipping), does not sufficiently decrease (non-dipping), or even increases instead (reverse dipping). Studies indicated that people with an abnormal nocturnal dipping are at a higher risk of developing target-organ damage, including damage in the optic nerve head.^1,8^ The introduction of ambulatory BP monitoring enabled the continuous assessment of BP over a 24-h period, providing detailed insights into the relationship between glaucoma and low BP.^47,48^ Despite some discordance in literature, most studies concluded that nocturnal hypotension and extreme nocturnal BP dipping are risk factors for the development and progression of open-angle glaucoma (cfr. Table 2). Patients with a nocturnal MAP drop greater than 10 mm Hg or 10% are at a higher risk for visual field progression.^6,12,15,37,47,49–53^ In addition, nocturnal BP decrease seems to be accompanied by IOP increase (due to the supine resting position) resulting in reduced OPP at night. When extremes of both phenomena occur simultaneously, OPP drops substantially resulting in short-term ischemia of the optic nerve head and significant risk for glaucoma progression.^54^ Therefore, nighttime could be considered as a critical period for glaucoma patients.^8,14,19^

**Table 2:** Nocturnal dipping and hypotension

		Type of study	Study population	Patients	Eyes	Men/ women	Country/ Ethnicity	Age	Systemic AHT	Conclusion	Significance level
Topouzis et al.^26^	2013	Cross-sectional, population-based study	POAG, PXG and controls	2,261 (94 + 41 + 2,126)	2261	1240/1021	Greece	70.8 ± 5.8 years	Included	Borderline significant association between low DOPP and POAG (*P* = 0.059)	*P* < 0.05
Charlson et al.^53^	2014	Prospective, longitudinal study	NTG	85	166	28/57	United States	Average 65 years	Included	Cumulative nocturnal hypotension (>10 mm Hg under DMAP) predicts visual field loss (*P* < 0.02)	*P* ≤ 0.05
Pillunat et al.^47^	2015	Cross-sectional study	POAG and NTG	314 (147 + 167)	314	113/201	Caucasian	>40 years	Included	Over-dippers with systemic normotension (with or without AHT) had more visual field loss than over-dippers with systemic hypertension (MD = −16.6 dB vs. MD = −3.9 dB, respectively; *P* < 0.004)	*P* < 0.05
Bowe et al.^6^	2015	Systematic review and meta-analysis	POAG and NTG	n.a.	n.a.	n.a.	n.a.	28–85 years	n.s.	Nocturnal systolic or diastolic BP dips > 10% (no differentiation between physiological- or over-dipping) is a risk factor for progressive visual field loss in glaucoma (*P* < 0.001 and *P* = 0.009, respectively; OR 3.32 and 2.09, respectively)	*P* < 0.05
Lee et al.^52^	2015	Longitudinal, retrospective, observational study	Untreated NTG	237	237	116/121	Korean	55.83 ± 9.33 years	Included	Significantly higher daytime or nighttime MAP and OPP variabilities were found in over-dipper NTG patients compared to non-dipper and dipper NTG patients. Baseline increased daytime MAP and OPP standard deviation significantly predicted future VFP in NTG	*P* < 0.05
Chiotoroiu et al.^51^	2015	Prospective observational and interventional study	OAG	45	90	n.s.	Romania	n.s.	n.s.	The dipper group (dips >10%) presented the most important progression of glaucoma (objectified by visual field and OCT) compared to the non-dipper (dips <10%) and arterial hypertension group	n.s.
Marjanovic et al.^13^	2015	Prospective, cross-sectional and observational study	NTG and NTG suspects (all arterial hypertension)	57 (37 + 20)	57	18/39	Serbia	≥50 years	Included	No statistically significant difference was found between NTG and NTG suspects in either DSBP or NSBP, nor in DDBP or NDBP. NTG patients had a lower nocturnal systolic and diastolic BP fall than NTG suspects	*P* < 0.05
Marjanovic et al.^48^	2016	Prospective, cross-sectional and observational study	POAG	114	114	78/36	Serbia	≥40 years	Included	There is a significant relationship between BP measurements (DSBP, DMAP and NSBP) and the RI in the OA in the dipper group. Retrobulbar blood flow parameters (EDV) are reduced in dippers (*P* < 0.001)	*P* < 0.0055
Jin et al.^49^	2017	Retrospective cohort study	POAG and NTG	106 (34 + 72)	106	56/50	Korean	POAG: 59.14 ± 10.18 years NTG: 55.88 ± 11.23 years	Excluded	Nocturnal BP dip (systolic and/or diastolic) and paracentral scotoma are significantly correlated (occurrence/progression) in early NTG but not significantly in early POAG. Large variations in BP affect the occurrence and progression of paracentral scotoma	*P* < 0.05
Kwon et al.^86^	2017	Prospective case-control study	NTG	349	698	168/181	Korean	55.9 ± 9.5 years	Included	Nocturnal over-dipping is a risk factor for the occurrence of ODH in NTG (*P* < 0.001), which is a significant prognostic factor for glaucomatous VFP in this study (*P* < 0.001). Increased variabilities of BP and OPP over 24 h are associated with a greater likelihood of glaucomatous VFP (*P* = 0.017 and *P* = 0.01, respectively)	*P* < 0.05
Kocatürk et al.^83^	2017	Prospective, randomized, case-control study	POAG, NTG and controls	129 (44 + 43 + 42)	n.s.	75/54	Turkey	63–72 years	Excluded	Systolic BP levels (24 h and nocturnal) and mPP values (24-h, day- and night-time) were significantly lower in NTG patients compared to POAG and controls. The number of extreme dippers was not significantly higher in the NTG compared to the POAG group. The physiopathology’s of NTG and POAG may vary	*P* < 0.05
Raman et al.^5^	2018	Prospective, longitudinal study	NTG	65	65	32/33	Malaysia	68.2 ± 9.8 years	Included	Baseline DBP and diastolic pressure parameters were significantly lower in patients who progressed (*P* < 0.05) and were significant factors in 5-year VFP among NTG patients. Low nocturnal DOPP is an independent predictor of glaucomatous VFP	*P* < 0.05
Melgarejo et al.^50^	2018	Observational, cross-sectional study	NTG, NTG suspects and healthy eyes	93	185	12/81	Hispanic	Mean 61.9 years	Included	Extreme dipping (dips >20%) of nocturnal BP levels, rather than nocturnal hypotension per se, increases glaucoma risk	*P* < 0.05
Kwon et al.^12^	2019	Prospective cohort study	NTG	119	119	51/68	Korean	54.15 ± 12 years	Included	Low nocturnal trough DBP (−10 mm Hg increased risk of VFP by 63.5%) and the duration and magnitude of the nocturnal dip (DBP dip area: 10 mm Hg × h increase of risk of VFP by 19.5%) at baseline are significant predictors of subsequent VFP	*P* < 0.05
Yoshikawa et al.^2^	2019	Observational, cross-sectional study	Glaucoma (POAG, PACG, SG and EG) and healthy controls	817 (109 + 708)	817	391/426	Japan	Glaucoma: 71 ± 11.2 Controls: 70.8 ± 6.8 years	Included	Significant association between glaucoma and increased NSBP (*P* = 0.001)and the non-dipper pattern of BP (*P* < 0.001), independent of known risk factors	*P* < 0.05
Karadag et al.^9^	2019	Observational study	POAG and PXG	18 (10 + 8)	n.s.	10/8	Turkey	POAG: 57.5 ± 8.5 years PXG: 67.3 ± 6.2 years	Excluded	In both groups, nighttime IOP was significantly higher than the daytime values. Nighttime SBP and DBP were significantly lower than the daytime values	*P* < 0.05
Baek et al.^58^	2020	Retrospective cohort study	NTG	102	102	37/65	South Korea	62.3 ± 14.1	n.s.	Fluctuations of DBP in 24-h BP, diurnal IOP fluctuations and ODH were significantly associated with NTG progression	*P* < 0.05
Yilmaz et al.^4^	2020	Retrospective case-control study	POAG and controls	75 (35 + 40)	n.s.	30/45	Turkey	POAG: 65.03 ± 14.56 years Controls: 59.98 ± 14.40 years	Included	The NSBP, whole day SBP and mean DBP were significantly lower in patients with POAG. Daytime, nighttime and whole day SBP were identified as independent risk factors for developing POAG in the multiple regression analysis. Hypotension is more significant in the etiopathogenesis of POAG	*P* < 0.05
Lee et al.^37^	2020	Retrospective cohort study	NTG	166	166	n.s.	South Korea	56.3 ± 15.3 years	Included	Patients with a minimum SBP ≤107 mm Hg showed more peripapillary RNFL thinning (*P* < 0.001) and patients with a minimum DBP ≤63 mm Hg had more progression of macular GCIPL thinning (*P* < 0.001)	*P* < 0.05
Leet et al. ^60^	2020	Retrospective study	NTG	110	220	48/62	South Korea	Average 56.75 years	Included	Extreme dipping (dips >20%) and arterial hypertension were independent predictors of VFD (*P* = 0.048 and *P* = 0.045 respectively)	*P* < 0.05
Shin et al.^59^	2021	Observational, cross-sectional study	NTG	88	88	35/53	South Korea	56.0 ± 12.4	Included	If choroidal microvascular drop-out was present on angiography-OCT, the worse the glaucoma severity (OR 0.786) and the more nighttime dips (OR 1.951)	*P* < 0.034
Melgarejo et al.^64^	2021	Observational, cross-sectional study	NTG	93	93	12/81	South America	61.9 ± 12.9	Included	24-h reading-to-reading mean arterial pressure (MAP) variability relates to glaucomatous optic neuropathy (OR, 1.93; 95% CI, 1.10-3.41) regardless the absolute MAP level	*P* < 0.05
Melgarejo et al.^66^	2022	Observational, cross-sectional study	NTG and POAG	93 and 48 OAG cases matched with 48 healthy contros	93 and 96	12/81 and 40/56	South America and Europe	61.9 ± 13.3 and 63.2 ± 11.9	Included	Dips rather than increases in the 24-h MAP level associates with increased risk of POAG (OR ranged from 2.25 to 3.39; 95% CI ranged from 1.31 to 8.46)	*P* < 0.05
Jammal et al.^54^	2022	Observational, retrospective longitudinal study	Glaucoma suspect, POAG, other	3,976	7,501	1577/2399	United States	64.5 ± 12.5	Included	Lower MAP and diastolic arterial pressure associates with faster rates of RNFL loss	
Melgarejo et al.^65^	2022	Observational, retrospective longitudinal study	POAG	110	110	65/45	Europe	68.5 ± 10.8	Included	Progression of functional glaucomatous optic damage is associated with high variability and extreme dips in the diurnal MAP while structural damage seems more vulnerable to nocturnal hypotension	

**Abbreviations**: OAG: open-angle glaucoma; POAG: primary open-angle glaucoma; NTG: normal-tension glaucoma; PACG: primary angle-closure glaucoma; SG: secondary glaucoma; EG: exfoliation glaucoma; PXG: pseudoexfoliation glaucoma; AHT: antihypertensive treatment; BP: blood pressure; SBP: systolic blood pressure; DBP: diastolic blood pressure; DSBP: daytime systolic blood pressure; DMAP: daytime mean arterial pressure; NSBP: nighttime systolic blood pressure; MD: mean deviation; RI: resistivity index; OA: ophthalmic artery; EDV: end diastolic velocity; ODH: optic disc haemorrhage; RNFL: retinal nerve fiber layer; GCIPL: ganglion cell-inner plexiform layer; OCT: optical coherence tomography; MAP: mean arterial pressure; VFP: visual field progression; VFD: visual field defects; OPP: ocular perfusion pressure; DOPP: diastolic ocular perfusion pressure; IOP: intraocular pressure; n.s.: not specified; n.a.: not applicable

Given that the abovementioned cut-off values for nocturnal BP dipping, fall partly within the physiological range,^17^ a deficient autoregulation mechanism is very likely. A study by Melgarejo *et al*.^50^ suggested that an increase in glaucoma risk is more likely to be due to the extreme dipping (dips > 20%) of nocturnal BP independently of the overall nocturnal BP level. Low diastolic OPP would particularly influence OPP as it determines the perfusion pressure to organs, explaining why is considered an independent risk factor for open-angle glaucoma.^5,8,19,20,26,55–57^ One study reported that a diastolic OPP <35 mm Hg result in progression of glaucoma 2.3 times more likely.^5^ In a cross-sectional study including POAG and NTG patients, patients with nocturnal over dipping and diurnal systemic normotension (both treated and untreated) had more visual field loss (mean deviation = −16.6 dB, IQR: −18.9 to −2.7 dB) than patients with nocturnal over dipping and diurnal systemic hypertension (mean deviation = −3.9 dB, IQR: −6.2 to −1.9 dB). They concluded that at the time of a 24-h ambulatory BP monitoring, nocturnal dipping patterns, and diurnal BP means should be analyzed in function of each other.^47^ Two other studies stated the importance of taking the cumulative nocturnal hypotension, the duration and magnitude of the nocturnal dip into account.^12,53^

Two study groups defined a comparable safety range for the nocturnal BP. Pillunat *et al*.^47^ proposed the Dresden safety range for nocturnal MAP in POAG ranging between 65 and 90 mm Hg. Kwon *et al*.^12^ defined optimal values of trough diastolic BP at night between 60 and 70 mm Hg. Patients with controlled IOP and a nocturnal BP within these safety ranges have slower progression rates than patients below this optimal value or might not be expected to progress at all.

In NTG patients, the association between nocturnal BP and glaucoma damage seems to offer particular insights. A retrospective study from 2017 found that nocturnal dipping (average amount of nocturnal decrease of BP) and large variations in systolic BP accorded to higher incidence of paracentral scotoma in early NTG patients.^49^ In their prospective case–control study, Kwon *et al*. surmised an IOP-unrelated mechanism of progression. They concluded that nocturnal dipping exerts its effect on glaucomatous visual field progression through the occurrence of optic disc hemorrhages.^15^ This was supported by a retrospective study from 2020, that found a significant association between optic disc hemorrhages and fluctuations of diastolic BP and diurnal IOP on one side, and a greater probability of NTG disease progression on the other side.^58^ Miscellaneously, higher percentages nighttime diastolic BP dips and more severe glaucoma were reported in NTG patients with choroidal capillary drop-out on angiography-OCT.^59^

##### Daytime hypotension

A 2020 retrospective cohort study found that minimum daytime systolic BP and diastolic BP could be, similar to nocturnal dipping or nocturnal hypotension, a potential risk factor for structural glaucomatous progression. Patients with a minimum systolic BP ≤107 mm Hg showed more peripapillary retinal nerve fiber layer thinning (*P* < 0.001) and patients with a minimum diastolic BP ≤63 mm Hg had more progression of macular ganglion cell-inner plexiform layer thinning (*P* < 0.001).^60^ These findings reinforce the importance of maintaining a minimal daytime BP level instead of an overall mean threshold level.

Jammal *et al*.^54^ report a significant faster rate of RNFL loss in glaucoma patients with lower mean BP, systolic BP or diastolic BP with and without antihypertensive treatment after correction for age, gender, race, glaucoma diagnosis, CCT, follow-up time, and baseline RNFL thickness in the Duke Glaucoma Registry.

##### Abnormal BP variability over 24-h

Ambulatory BP monitoring permits the quantification of reading-to-reading BP variability over 24-h. In the hypertension and cardiovascular fields, high variability in the 24-h BP increases the risk of cardiovascular diseases independently of the average 24-h BP level.^61^ High variability in the BP suggests impaired autonomous central nervous system mechanisms to maintain a constant BP level, which is the case in patients with diabetes, obesity, or previous cardiovascular diseases. Increased BP variability would not be regulated in eyes with glaucomatous damage as their autoregulatory mechanisms to maintain the ocular blood flow and supply would be impaired. Therefore, beyond the absolute level and nocturnal hypotension, it is hypothesized that high variability in the BP over 24-h could increase the risk of glaucoma damage by impaired OPP related to abnormal changes in the systemic BP.

Compared to the cumulative evidence on nocturnal hypotension, few studies have addressed the potential role of 24-h BP variability and glaucoma risk. In 237 patients with NTG, Lee *et al*.^52^ documented that patients with nocturnal BP dipping greater than 20% had significantly increased reading-to-reading daytime BP variability and OPP (defined as by Bill *et al*.^62^) compared to normal dippers or non-dippers. Moreover, an increased daytime MAP or OPP variability predicted the progression of visual field defects. Similar findings have been replicated in case–control studies of patients with NTG,^58,63^ with additional documentation of fluctuations in the diastolic OPP related to the progression of glaucoma damage.^58^ In a study including 93 participants and 23 cases of open-angle glaucomatous damage, researchers reported that the association between high 24-h MAP variability and glaucoma risk is independent of the 24-h MAP average level.^64^ Even more, high 24-h MAP variability related to higher glaucoma progression.^65^

From a pathophysiological perspective, it is hypothesized that drops in the BP due to high variability is what leads to impaired OPP. Apart from nocturnal hypotension, the quantification of 24-h BP variability relies on indexes such as standard deviation, coefficient of variation, or variability independent of the mean. Each of these indexes gives an absolute number usually reported in publications with the symbol “±.” This refers to how far apart data points (e.g., BP recordings) are from the center of the distribution (e.g., 24-h BP average). The extrapolation of this definition to the pathophysiology of glaucoma suggests that the association between variability and glaucoma needs to be addressed. In this regard, the Leuven research group conducted a study to test the hypothesis that the association between high 24-h MAP variability and glaucoma risk was driven by sporadic drops in the MAP rather than peaks.^66^ To test this hypothesis, they equitably quantified the five largest drops and peaks in the MAP over 24 h (*n* = 94 and *n* = 96). Dips rather than peaks in the 24-h MAP related to open-angle glaucoma damage. This could be explained by the fact that patients with normal but highly variable BP are more likely to reach lower OPP repeatedly, and secondly, because patients with high BP exhibit higher BP variability, being more likely to excessively drop in BP, reducing OPP. With this in mind, physicians should consider that the majority of glaucoma patients have normal or high BP—the last might explain why arterial hypertension relates to glaucoma risk as depicted in the first paragraph.^7,31–43^ Antihypertensive medication should therefore aim to stabilize BP variability, avoiding extreme dips in the BP, while ensuring nocturnal BP is normal. This in turn should decrease the risk of glaucoma associated with sporadic or constant low BP.

#### Systemic antihypertensive medication

Studies investigating the role of antihypertensive medication in glaucoma are often not corrected for the presence or severity of existing hypertension and yield contradictory conclusions (cfr. Table 3).^17,67^

**Table 3:** Antihypertensive medication

		Type of study	Study population	Patients	Eyes	Men/ women	Country/ Ethnicity	Age	Antihypertensives	Conclusion	Significance level
Topouzis et al.^68^	2006	Cross-sectional population-based epidemiologic study	Non-glaucoma population	232	232	138/94	Greece	Mean 71 years	Included but n.s.	A DBP<90 mmHg resulting from AHT is associated with increased optic disc cupping and decreased rim area.	*P* < 0.05
Suïc et al.^57^	2015	Prospective cohort study	Glaucoma patients with treated systemic hypertension	64	n.s.	36/28	Croatia	♂: 65,32 years ♀: 62,45 years	Included	Statistically significant lower DBP in the progressive group.	*P* < 0.05
Horwitz et al.^69^	2017	Registry database study	Danish glaucoma patients with hypertension	41,235	n.a.	n.s.	Denmark	40-95 years	Antiadrenergics Diuretics Vasodilators Beta blockers CCB A2RBs ACE inhibitors	Antihypertensive medication seems to delay the onset of developing glaucoma but does not necessarily reduces the immediate risk. A greater protective effect was found depending on the cumulative number of different antihypertensive drugs.	*P* < 0.05
Höhn et al.^74^	2017	Population-based, prospective, observational cohort study	Non-glaucoma population	13,527	n.s.	6,849/ 6,678	Germany	Mean 54,3 years	Peripheral vasodilators Diuretics Beta blockers CCB RAB ACE inhibitors ARB Nitrates Other AHT medication	Non-selective beta blockers showed a statistically non-significant trend of slightly lower IOP. All the other cardiovascular medication did not show an association.	*P* < 0.0038
Zheng et al.^42^	2018	Database study	POAG and controls	36780 (6,130 + 30,650)	n.a.	17,847/ 3,166	United States	Mean 72 years	Beta blockers CCB A2RB ACE inhibitors Loop diuretics	CCB (mainly Amlodipine) were associated with POAG requiring filtration surgery. Beta blockers had a protective association with POAG.	*P* < 2.3 × 10^−5^
Siddiqui et al.^73^	2019	Retrospective, long-term, case-control analysis	POAG	111	n.a.	48/63	Caucasian + Hispanic + others	Mean 70 years	ACE inhibitors ARB Beta blockers Thiazides Loop diuretics CCB	There was no significant impact from systemic antihypertensive medication on IOP reduction after topical prostaglandin initiation. Systemic antihypertensives use was not correlated with nonresponse to prostaglandin therapy.	n.s.
Wang et al.^77^	2019	Cohort study	OAG	n.s.	n.a.	n.s.	United States	n.s.	A2RB Thiazide diuretics CCB	There is more rapid progression to glaucoma filtration surgery in patients taking CCB as compared with thiazides. This relationship was not found for the other drugs investigated.	*P* < 0.05
Pappelis et al.^70^	2019	Retrospective cohort study	POAG cases and suspects	362 (250 + 112)	362	185/177	Caucasian	Mean between 55-62 years	Diuretics ARB ACE inhibitors CCB Beta blockers	None of the systemic medications were associated with POAG VFP. A2RBs significantly delayed progression in older patients. ACE inhibitors and A2RBs were significantly associated with a lower risk of POAG suspect conversion.	*P* < 0.05
Chong et al.^67^	2020	Population-based, cross-sectional study	Data form the Singapore Epidemiology Eye Diseases Study	4,699	n.s.	2292/2407	Multi-ethnic Asian	58.8 ± 8.5 years	ACE inhibitors A2RB CCB Diuretics Beta blockers	The use of antihypertensive medication, especially ACE inhibitors and diuretics, were significantly associated with thinner RNFL and GCIPL. A greater number of antihypertensive medications was also associated with thinner RNFL and GCIPL.	*P* < 0.05
Hu et al.^76^	2021	Prospective cohort study	NTG	20	20	17:3	China	53.7 years between 32–68	Nimodipine	Nimodipine increased superficial macular capillary vessel density	*P* ≤ 0.04
Funk et al.^34^	2022	Retrospective case-control study	NTG and controls	277/ 277	n.s.	236 /318	United States	69.5 and 68.9	ACE inhibitors A2RB CCB Diuretics Beta blockers	Numerous vascular risk factors were associated with NTG, including systemic hypotension and hypertension. The use of angiotensin-converting enzyme inhibitor or calcium channel blocker were associated with NTG.	*P* < 0.05
Jammal et al.^54^	2022	Retrospective, longitudinal cohort study	Duke Glaucoma registry of glaucoma or suspected glaucoma	3,976	7501	1,580/2,399	United States	64.5 (IQR, 57.3–72.9)	ACE inhibitors A2RB CCB Diuretics Beta blockers	Combination of low mean arterial pressure with low diastolic blood pressure with high intraocular pressure increases the risk of progression of glaucoma damage.	*P* ≤ 0.007
Lee et al.^71^	2022	Retrospective, longitudinal cohort	OAG	20,815	n.s.	7,336/13,479	United States	86 between 40 and 80+	ACE inhibitors A2RB CCB Diuretics Beta blockers	Low BP was associated with the development of OAG whereas treatment for lowering BP was not.	*P* = 0.022

**Abbreviations**: OAG: open-angle glaucoma: POAG: primary open-angle glaucoma; AHT: antihypertensive treatment; DBP: diastolic blood pressure; IOP: intraocular pressure; CCB: calcium channel blockers; ARB: angiotensin receptor blockers; A2RBs: angiotension II receptor blockers; ACE inhibitors: angiotensin-converting enzyme inhibitor; RAB: Renin-angiotensin blockers; VFP: visual field progression; n.a.: not applicable; n.s.: not specified

The Thessaloniki Eye Study reported that iatrogenic DBP <90 mm Hg leads to increased cupping and decreased rim area compared to spontaneous DBP <90 mm Hg.^68^ Hence, some postulate that the (over)treatment of hypertension, rather than the disease itself, is a significant modifier of glaucoma.^11,56^ Aggressive decrease in the BP due to antihypertensive treatment could potentially lead to low DBP and consequently low OPP.^56,57,69,70^ Thus, when treating systemic hypertension, an increase in glaucoma risk could exist if OPP decreases.^11^ Some studies found a correlation between progression and the number of antihypertensive agents^54,67^ whereas another did not.^71^ Ocular blood flow could potentially be improved in hypertensive patients with drug-induced low nocturnal BP by adapting their medical regime.^57,72^ For instance, changing the time of medication intake from the evening to morning.^4,73^

On the other hand it is also believed that undertreatment of hypertension may influence the disease progression of hypertensive glaucoma patients and that some antihypertensive drugs may have protective effects on glaucoma development.^1,14^ A Danish registry database study reported that antihypertensive medication seems to delay glaucoma onset but not necessarily reduce the immediate risk thereof. They also found a greater protective effect proportional to the cumulative number of different antihypertensive drugs.^69^

The effect of systemic antihypertensive medication on glaucoma risk can be either IOP- or non-IOP related.^3,74^ It has been established that systemic β-blockers, especially non-selective types, have a lowering effect on IOP. However, in the Gütenberg Health Study, Höhn *et al*. could not detect a significant trend of lower IOP (selective BB: −0.12 mm Hg; non-selective BB: −0.7 mm Hg) in non-glaucoma subjects. This finding was attributed to a long-term “drift” effect.^74^

Regarding calcium channel blockers (CCB), mixed findings have also been reported. On one hand, it has been suggested that CCB delay visual field deterioration (possibly due to a neuroprotective effect).^3,14,75^ Hu *et al*.^76^ documented that nimodipine benefits patients with NTG by increasing the macular capillary vessel density evaluated on OCT-angiography. On the other hand, studies have found that CCB increase the risk of POAG after controlling for systemic hypertension (OR 1.70 *P* = 0.03).^34^ Zheng *et al*. found that CCB, especially amlodipine, were the most significant drug class to be associated with a 26% risk increase of POAG (having had at least one glaucoma procedure) (OR 1.26, 95% CI: 1.18–1.35). No dose-response relationship was identified. β-blocker use was associated with a 23% lesser incidence of POAG (OR 0.77; 95% CI: 0.72–0.83). No association between POAG and the use of loop diuretics or angiotensin-converting enzyme (ACE) inhibitors could be established.^42^ A more recent study underlines the relative higher accordance between CCB and filtration surgery in POAG compared to thiazides. Other drugs investigated in this study, such as angiotensin-II receptor blockers (ARB), had no significant association with POAG progression.^77^ Caution must be made since these findings often do not correct for the existence of concomitant hypertension.

A retrospective study investigating a cohort from the Groningen Longitudinal Glaucoma study observed a good and highly significant interaction between age and angiotensin-II receptor blockers in relation to glaucoma progression. This suggests a higher benefit of ARB on glaucoma progression in elderly individuals. A significant association between ACE inhibitors, ARB and lower suspect POAG was also reported.^70^ On the other hand, a large cross-sectional population-based study in a multi-ethnic Asian population found that patients using antihypertensive medication, particularly ACE inhibitors and diuretics, had significantly thinner retinal nerve fiber layers and ganglion cell-inner plexiform layers.^67^

Synergistic or antagonistic effects of certain antihypertensive agents with certain topical glaucoma therapies have been considered. The addition of a topical β-blocker may not induce a significant reduction in IOP if the patient has already been prescribed an oral β-blocker. Moreover, all drug combinations could increase the risk for adverse effects.^3^ A long-term case–control study investigating how systemic antihypertensive medications influence the change in IOP after initiating prostaglandin drop therapy did not find a significant impact of antihypertensive medication on the IOP-reduction after topical prostaglandin initiation.^73^

#### Autoregulation

In healthy eyes, retinal blood flow is autoregulated and a relatively constant blood flow is maintained despite changes in the local metabolic environment and changes in OPP.^19,22,44^ Constant perfusion can be assured within the range of approximately 20 mm Hg around the patients usual MAP.^53^ When OPP falls out of this range, autoregulation fails.

Some glaucoma patients have impaired autoregulation of ocular blood flow (cfr. Table 4). In these patients, ocular blood flow instability may predispose the optic disc structures to ischemia-reperfusion damage. It has been documented that both tails of the arterial BP distribution relates with impaired autoregulatory mechanism in the eyes to maintain an adequate blood flow and supply.^78^ As previously mentioned, hypertensive glaucoma patients also have, microvascular damage which further disrupts autoregulation mechanisms and increases susceptibility to glaucoma progression. Studies reporting nocturnal hypotension and increased variability of BP and OPP as risk factors for glaucoma also supported that functional vascular dysregulation could be involved in the pathogenesis of glaucoma.^13,22,24,49,79,80^ This hypothesis may also explain why lowering IOP beyond a critical value is sufficient to restore OBF in certain patients, but may be inadequate as a treatment in patients with significant autoregulatory dysfunction.^14^

**Table 4:** Autoregulation

		Type of study	Type of glaucoma/ population	Patients	Eyes	Men/ women	Country/ Ethnicity	Age	Systemic AHT	Conclusion	Significance level
Modrzejewska et al.^80^	2015	Case-control study	POAG and controls	110 (56 + 54)	110	n.s.	Poland	Mean 68 years	Excluded	POAG was associated with significantly higher arterial BP, increased resistance indices and significantly lower OPP, DOPP and blood flow velocities. Vascular factors could have a vasoconstrictive role in the glaucomatous endotheliopathy.	*P* ≤ 0.05
Binggeli et al.^82^	2018	Retrospective study	Glaucoma patients	57	n.a.	22/35	Switzerland	17–92 years	n.s.	Patients with vascular dysregulation had on average lower systolic and diastolic BP.	*P* < 0.05
Lindemann et al.^79^	2018	Prospective clinical validation study	POAG, NTG and controls	146 (37 + 27 + 82)	n.a.	74/72	Germany	POAG: 69.9 ± 9.9 years NTG: 69.8 ± 8.5 years Controls: 60.7 ± 15.9 years	Included	There is a higher occurrence of BP and HRV in NTG which indicates impaired autonomic cardiovascular dysregulation.	*P* < 0.01
Cao et al.^84^	2018	Case-control study	POAG, NTG and controls	73 (18 + 19 + 36)	n.a.	60/13	Australia	50–80 years	Excluded	Both NTG and POAG manifest some systemic autonomic dysregulation to carbohydrate ingestion and postural change, but the characteristics of the dysregulation may differ between the two subtypes.	*P* < 0.05
Kiyota et al.^24^	2020	Prospective-longitudinal study	OAG	16	28	7/9	Japan	55.7 ± 13.4 years	Included	Weaker ONH tissue vasoreactivity to systemic hyperoxia was, among other things, associated with rapid VFP.	*P* < 0.05
Asefa et al.^81^	2020	Prospective population-based cohort study	Primary glaucoma and controls	86,841	n.s.	35,459/ 51,382	The Netherlands	Glaucoma: 53.4 ± 12.7 years Controls: 46.1 ± 12.6 years	Included	Low HRV (a measurement for autonomic modulation of the heart), high BP, hypertension and antihypertensive medication were associated with glaucoma.	*P* ≤ 0.05
Papellis et al.^78^	2021	Prospective cohort study	Healthy subjects	96	96	58/38	The Netherlands	Average ranged from 55.9 to 57.2 years old	Included	Inner retinal thinning was associated with low but also high BP levels, and with ineffective autoregulation.	*P* ≤ 0.045

**Abbreviations**: NTG: normal-tension glaucoma; POAG: primary open-angle glaucoma; BP: blood pressure; OPP: ocular perfusion pressure; DOPP: diastolic ocular perfusion pressure; ONH: optic nerve head; VFP: visual field progression; HRV: heart rate variability; n.a.: not applicable; n.s.: not specified

Some glaucoma patients also present with features of a more generalized vascular dysfunction. Lindemann *et al*.^79^ found a higher occurrence of impaired autonomic cardiovascular dysregulation in NTG. The Lifelines Cohort Study found that low heart rate variability, a measurement for autonomic modulation of the heart, was associated with glaucoma.^81^ Binggeli *et al*. used nailfold capillaroscopy with cold provocation to examine the relation between BP and vascular dysregulation in glaucoma patients. Their study revealed that patients with vascular dysregulation have on average lower systolic and diastolic BP. Both vascular dysregulation and low BP are core elements of Flammer syndrome, of which the prevalence is higher in NTG.^82^

Kocatürk *et al*. assessed the BP charts of healthy patients and glaucoma patients. They noted that the systolic and diastolic BP graphs of glaucoma patients seemed blunted compared to those of healthy patients, especially in the early morning. This suggests that early morning blunted sympathetic activity may play a role in the pathophysiology of glaucoma.^83^

Another study investigated the autonomic regulation to carbohydrate ingestion and postural change in 19 NTG, 18 POAG and 36 control patients, age and gender matched. They concluded that both NTG and POAG manifest some systemic autonomic dysregulation, but that the characteristics of the dysregulation may differ between the two subtypes.^84^

## DISCUSSION

This systematic review provides an up-to-date evaluation of the interplay between systemic BP and glaucoma progression. Although there is some discordance, there appears to exist a bimodal, U-shaped relation between BP and glaucoma progression.^20,57^ Both low and high BP relate to lower RNFL and ganglion cell thickness, especially in the absence of adequate autoregulation on both sides.^78^ Low BP, whether intrinsic or drug-induced, leads to low OPP and, in the absence of sufficient autoregulation, possibly to ischemia of the optic nerve head. High BP is associated with higher IOP, higher BP variability and microvascular disease which in turn leads to lower perfusion. Next to nocturnal over-dipping, this review highlights high BP variability as an additional glaucoma progression risk factor.^50,64–66,85^ Recent evidence indicated that an increased reading-to-reading variability and drops in MAP over 24-h related to progression visual field defects in patients with POAG,^85^ especially during the daytime.^65^ The relation between antihypertensive treatment and glaucoma—in studies often uncorrected for the presence or severity of hypertension—is insufficiently understood, mostly given the bias of existing refractory hypertension. Lastly, the correlation of glaucoma with nocturnal BP dipping has been established and systemic hypotension should be ruled out in case of glaucomatous progression, especially in patients with normal or well-controlled IOP.^4^ A simple, non-invasive method such as 24-h ABPM proves to be a valuable tool in BP assessment.^14,37,83^ Intensive BP treatment and the time of antihypertensive drug intake could increase the effect of nocturnal dipping depending on the patients susceptibility.^86^ If nocturnal hypotension is detected, change in pharmacological treatment might be considered. Although morning intake would theoretically reduce the risk of nocturnal dipping, the Hygia and MAPEC trials point to a more pronounced reduction of cardiovascular mortality associated with nighttime dosing.^4,73,87^

The clinical implementation of the findings of the SPRINT trial, STEP trial and the most recent guidelines concerning the treatment of systemic hypertension may have a key role in the management of glaucoma the coming years.^11,88,89^ The trial demonstrated that treating arterial hypertension to a target of less than 120 mm Hg reduced cardiovascular events and the overall risk of death in all hypertensive patients.^88^ Following those findings, the 2017 ACA/AHA hypertension guidelines redefined office hypertension as a systolic BP of 130 mm Hg or higher or DBP of 80 mm Hg or higher.^90^ These stricter targets broaden the group of patients needing antihypertensive treatment and will increase the number of patients with coexisting systemic hypertension and glaucoma. In this group, glaucomatous progression due to medication-induced hypotension is likely to become more frequent, despite well-controlled IOPs, causing a clinical dilemma between cardiologists and ophthalmologists. Although reducing cardiovascular mortality takes precedence over preserving vision, the impact of visual impairment on psychosocial well-being and quality of life should also be considered. A balance between quality adjusted life years, disability-adjusted life years, and years of life lost must be considered when evaluating patients with glaucoma and concomitant hypertension. A hypothetical approach could give priority to the independent increase of diastolic BP, given some—older—studies pointing to systolic BP as only modifier of cardiovascular risk and the proven importance of diastolic BP in glaucoma.^91,92^ Recently some additional evidence regarding the choice of antihypertensive in association with glaucoma risk has been published. A retrospective study on antihypertensive use in 31,170 glaucoma vs. non-glaucoma participants with arterial hypertension did not show higher incidence of glaucoma in the groups on single diuretics, ACE inhibitors or β-blockers, but did show higher odds ratios in the groups on ARB monotherapy, CCB monotherapy, and various combination treatments.^93^ Additionally, a study on glaucoma entries in the UK biobank (*n* = 427,480) led to the conclusion that CCB treatment is associated with a 1.39 odds ratio (*P* = 0.001) on having glaucoma.^94^ The deleterious effect of CCB on glaucoma incidence was in line with two other recent meta-analyses.^95,96^ A possible causal mechanism of this CCB effect is that impairment of autoregulation might result in no further pharmaceutical dilation in affected zones and that dilation of other—more healthy—capillary beds shunt away blood to unaffected areas.^96,97^ Previously, CCBs (more specifically nifedipine) and ACE inhibitors have been argued beneficial in the discussion around endothelial dysfunction and Flammer syndrome. This highlights the necessity to adjust for vascular comorbidity as these drugs might be used in patient groups that are prone to glaucoma due to a vascular cause.^98^ This also prompts the idea for studies designed to further quantify autoregulation and retinal vascular response before and after start of antihypertensive medication including the different classes of CCBs.

Regarding β-blockers, one meta-analysis also points to a decreased glaucoma incidence (OR 0.83 [0.75–0.92]). However, only in one of the included studies, adjustment for covariates as BMI, smoking status, age, gender, and incident hypertension was executed. In this study, the lower OR for β-blockers and glaucoma incidence was barely significant (0.91 [0.83–0.99]).^95,99^

Regarding ACE inhibitors, only two studies provided evidence against its use. Chong *et al*.^67^ found that ACE inhibitors (and diuretics) were associated with more RNFL and GCL thinning after adjustment of covariates. Langman *et al*.^100^ report higher incidence of POAG in this group both in current as past intake, which brings the authors to point towards uncontrolled hypertension rather than the class of medication as culprit.

Taken together the authors speculate that as first line treatment for hypertension in glaucoma patients thiazides and/or to a lesser extent ACE inhibitors or β-blockers, depending on concomitant comorbidities as heart failure, lung or kidney disease, could be considered (rather than CCBs). However, validation studies are needed to support such clinical recommendations.

Ophthalmologists do not commonly treat hypertension and professionals who do, haven’t established preferred practice patterns on the management of arterial BP in their glaucoma patients.^17^ The importance of arterial BP in glaucoma management has also not yet been addressed in recent studies or most guidelines on the diagnosis and treatment of arterial hypertension.^101^ Glaucoma featured for the first time in the new hypertension guideline released in 2023 by the European Society of Hypertension. Most of our recommendations below are in line with this guideline.^102^ Of note, the recommendation puts β-blockers forward as mainstay treatment in hypertensive patients with glaucoma, based on one study and an possible IOP-lowering effect.^42,103,104^ Although, β-blockers seem at least harmless in glaucoma (if not potentially beneficial also due to their IOP-lowering effect), in terms of mortality reduction, β-blockers seem to be inferior to thiazides or ACE inhibitors and β-blockers lead to greater DBP reduction than thiazides.^105–107^ Therefore, this recommendation may be reconsidered in a future update of the Hypertension Guidelines.

Further studies investigating BP targets and their impact on glaucoma incidence and progression are needed. Additional sub-analyses studying the effect of antihypertensive drugs on glaucoma incidence and progression also merit to be investigated more.

Limitations of this review are methodological in nature and inherent to the represented research. Only papers in English were included, OPP and BP parameters are heterogeneously reported, and a lot of studies do not adjust for continuous BP levels, or even the mere presence of arterial hypertension, nor other covariates.

**Figure 1.**
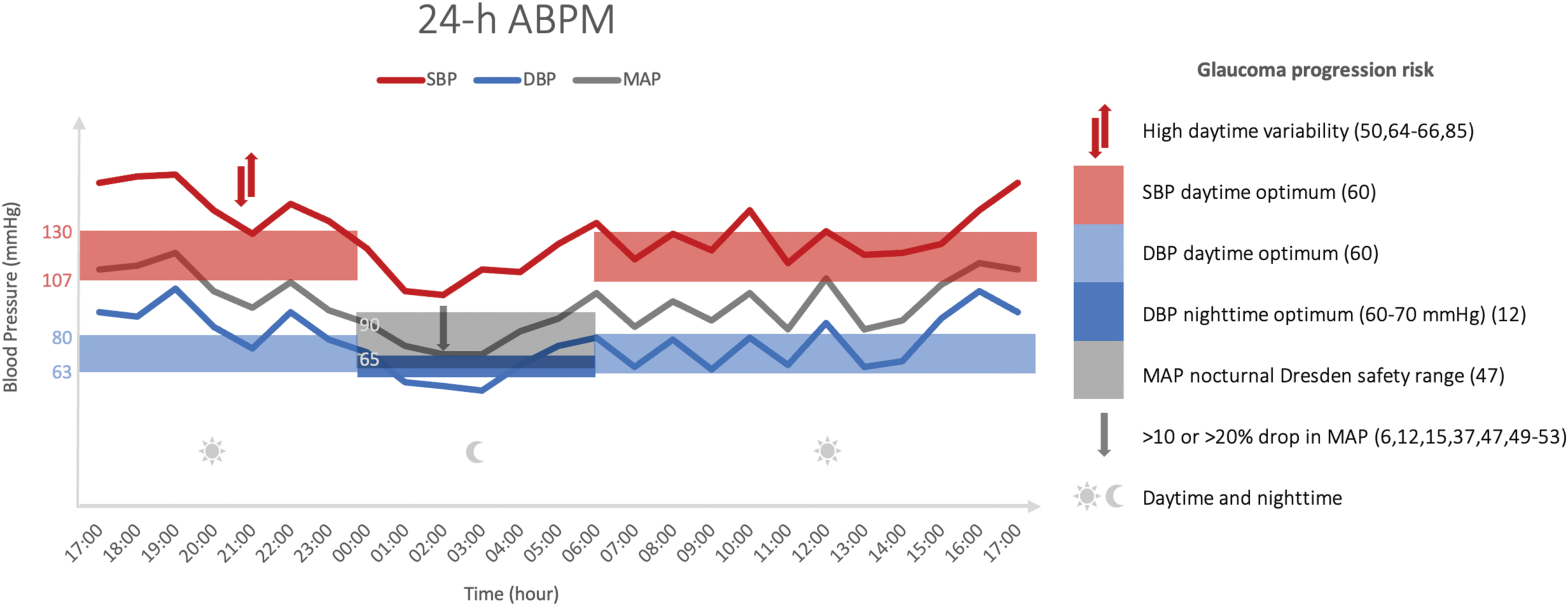
Figure of a simplified, individual example of a 24-h ABPM with overlay of recommended BP levels. The recommendations are based on the cited references that are also mentioned in the manuscript text, where the daytime upper limits are derived from the ACA/AHA hypertension guidelines.^90^ This includes the daytime systolic optimum between 107 and 130 mm Hg,^60^ the daytime diastolic optimum between 63 and 80 mm Hg,^60^ the nocturnal MAP Dresden safety range between 65 and 90 mm Hg^47^ and the nocturnal diastolic optimum between 60 and 70 mm Hg.^12^ High BP variability features in this review as glaucoma progression risk factor, however clear cut-off values have not been put forward yet.^50,64–66,85^ This patient exhibits all mentioned risk factors for glaucoma progression except overall nocturnal MAP that is still in the Dresden safety range.^47^ These overlays could visually aid the clinician in evaluating 24-h ABPMs. ABPM, ambulatory blood pressure measurement; DBP, diastolic blood pressure; MAP, mean arterial pressure; SBP, systolic blood pressure.

Based on our systemic literature review, we propose some clinical recommendations for the management of glaucoma patients with concomitant systemic hypertension, summarized in Figure 1, and some research recommendations for future studies on this topic.

### Clinical recommendations

- If a patient presents with glaucomatous progression despite normal or well-controlled IOP, 24-h ABPM is useful to rule out nocturnal systemic hypotension or high BP variability.
- In glaucoma patients with concomitant systemic hypertension a low threshold to perform a 24-h ABPM is justified.
- Antihypertensive adjustment should be based on 24-h ABPM. Repeated 24-h ABPM is recommended after medication changes.
- Diagnosed nocturnal (over-)dipping or high BP variability in a glaucoma patient should prompt a collaborative treatment strategy between the treating physician and ophthalmologist.
- In case of high daytime BP fluctuation, high (to normal) BP and relatively absent nocturnal dipping, intensification of the antihypertensive treatment could lower BP variability.
- In case of nocturnal (over-)dippers, morning dosing of antihypertensive medication could be considered (taking into account the pros and cons as discussed above).
- Up till now there is insufficient evidence to justify decrease of antihypertensive medication in case of merely nocturnal over-dipping.
- Currently available evidence suggests that CCB may not be recommended as first line treatment in patients with both glaucoma and arterial hypertension. Instead, thiazides and/or to a lesser extent ACE inhibitors might be more suitable. β-blockers might also be potentially beneficial in glaucoma. However, additional evidence is needed to support such clinical recommendations.

### Research recommendations

- Future research might benefit from BP data being presented continuously, and when categorized, being presented by different cut-off values to enable comparison.^108^
- Future research should correct for covariates, including the presence of hypertension, when comparing different types of antihypertensive medication.

## Supplementary Material

hpad111_suppl_Supplementary_Data
