# Supplementary material for: The Relevance of Arterial Blood Pressure in the Management of Glaucoma Progression: A Systematic Review
Source: Am J Hypertens. 2023 Nov 23;37(3):179–98. doi: 10.1093/ajh/hpad111 (PMC10906067; doi:10.1093/ajh/hpad111)
Supplement: hpad111_suppl_Supplementary_Data [file hpad111_suppl_supplementary_data.docx]

**Supplemental Data**

**Search** **strategy**

Pubmed

((((("Glaucoma"[Mesh] OR glaucoma*[tiab])) AND ("Arterial Pressure"[Mesh] OR arterial pressure* [tiab] OR arterial tension*[tiab] OR “artery pressure”[tiab] OR “intraarterial pressure”[tiab] OR "Blood Pressure"[Mesh] OR blood pressure*[tiab] OR "Hypertension"[Mesh] OR hypertensi*[tiab] OR "Hypotension"[Mesh] OR hypotensi*[tiab])) AND ("Disease Management"[Mesh] OR Management*[tiab] OR Monitoring[tiab]OR "Therapeutics"[Mesh] OR therap*[tiab] OR “treatment”[tiab] OR adapt*[tiab] OR change*[tiab] OR approach*[tiab] OR disease control*[tiab] OR risk*[tiab] OR progression*[tiab] OR visual field*[tiab]))) NOT (("Animals"[Mesh] NOT ("Animals"[Mesh] AND "Humans"[Mesh])))

*Filters*: Full text; English; Publication date from 2015/01/01

Embase

('glaucoma'/exp OR 'glaucoma*':ti,ab,kw)

AND

('arterial pressure'/exp OR 'arterial pressure*':ti,ab,kw OR 'blood pressure'/exp OR 'blood pressure*':ti,ab,kw OR 'hypertension'/exp OR 'hypertension':ti,ab,kw OR 'hypotension'/exp OR 'hypotension':ti,ab,kw)

AND

('disease management'/exp OR 'disease management':ti,ab,kw OR 'diseases management':ti,ab,kw OR 'management'/exp OR 'management*':ti,ab,kw OR 'monitoring'/exp OR 'monitoring':ti,ab,kw OR 'therapy'/exp OR 'therap*':ti,ab,kw OR 'adapt*':ti,ab,kw OR 'change'/exp OR 'change*':ti,ab,kw OR 'approach*':ti,ab,kw OR 'disease control'/exp OR 'disease* control*':ti,ab,kw OR 'risk'/exp OR 'risk*':ti,ab,kw OR 'visual field'/exp OR 'visual* field*':ti,ab,kw OR 'progression*':ti,ab,kw)

NOT

([animals]/lim NOT [humans]/lim)

*Filters*: English; 2015-2022

Web of Science

TS=("Glaucoma" OR glaucoma*)

AND

TS=("Arterial Pressure" OR arterial pressure* OR arterial tension* OR "artery pressure" OR "intraarterial pressure" OR "Blood Pressure" OR blood pressure* OR "Hypertension" OR hypertensi* OR "Hypotension" OR hypotensi*)

AND

​TS=("Disease Management" OR Management* OR Monitoring OR "Therapeutics" OR therap* OR "treatment" OR adapt* OR change* OR approach* OR disease control* OR risk* OR progression* OR visual field*)

*Filters*: English; last 5 years

Cochrane library

(arterial pressure):ti,ab,kw OR (blood pressure):ti,ab,kw

AND

(glaucoma):ti,ab,kw

**PRISMA flow diagram**

## Identification

Additional records identified through other sources
(n = 11)

Records identified through database searching
(n = 8681)

Full-text articles excluded, (full text not available, not in English, did not meet the inclusion criteria)
(n = 28)

## Included

## Eligibility

## Screening

Full-text articles assessed for eligibility
(n = 124)

Abstracts excluded
(n = 181)

Titles excluded
(n = 6276)

Abstracts screened
(n = 305)

Titles screened
(n = 6581)

Records after duplicates removed
(n = 6581)

**Supplementary figure 1. Flow chart of the systemic review.**

Included studies
(n = 81)
